# A Sealed Water Calorimeter for Measuring Absorbed Dose

**DOI:** 10.6028/jres.099.012

**Published:** 1994

**Authors:** Steve R. Domen

**Affiliations:** National Institute of Standards and Technology, Gaithersburg, MD 20899-0001

**Keywords:** absorbed dose, calorimeter, convective barrier, heat defect, thermistor, water

## Abstract

The NIST sealed water calorimeter is intended for direct measurement of absorbed dose to water. This calorimeter was used for a series of approximately 3700 measurements to investigate the so-called heat defect, that is, anomalous endothermic or exothermic effects caused by dissolved gases. The three systems investigated were “high-purity” water saturated with N_2_, H_2_, and mixtures of H_2_/O_2_. The repeatability of the measurements of absorbed dose rates for the ^60^Co teletherapy beam was studied with different water fillings and accumulated absorbed dose. Measurements with the H_2_/O_2_ system varied with accumulated absorbed dose. Based on the measurements and theoretical considerations, it appears that the H_2_-saturated system is the best choice for eliminating the heat defect. Measurements with both the N_2_- and H_2_-saturated systems are in good agreement with those determined with a graphite and graphite-water calorimeter (for which there is no heat defect).

## 1. Introduction

Absorbed dose^1^ is widely used to quantify the medical or biological use of ionizing radiation. The output of a radiation therapy accelerator is calibrated by determining the absorbed dose rate in water, which has been chosen as the standard reference material [1] because it has absorption and scattering properties similar to tissue. Traditionally, absorbed dose is determined from measurements with an ionization chamber and using a protocol [2], which gives the procedure along with numerous correction factors for determining the absorbed dose to water. It has been shown [3–5] that the most nearly direct approach is with a water calorimeter. In principle (when the heat defect is negligible), the absorbed dose at the measurement position is simply the product of the temperature rise and the specific heat capacity of water. Yet this direct approach was not considered possible because of technical difficulties [1].

The possible success of the water calorimeter was viewed mainly as involving two investigative phases [4] as to whether: (1) an accurate small temperature rise could be measured with a sufficiently good signal-to-noise ratio, and (2) the heat defect could be dealt with by its elimination or a correction applied for its effect. In the present work, these effects were investigated in three systems of “high-purity” water saturated with high-purity gases of (1) nitrogen, (2) hydrogen, and (3) mixtures of hydrogen and oxygen. They were investigated as a function of accumulated absorbed dose over long periods of time and with different water fillings.

The temperature rise was measured with two calibrated thermistors in opposite arms of a Wheatstone bridge to double the output signal. For negligible heat defects and changes in thermistor power, the absorbed dose *D* is:

$$D=(1/2)(\Delta R/R)|{\bar{S}}^{-1}|c,$$ (1)

where *D* is the absorbed dose,

1/2 is the result of using two thermistors to measure a temperature rise,
Δ*R/R* is the measured fractional change in the Wheatstone bridge balancing resistor,
$\left|{\bar{S}}^{-1}\right|$ is the absolute value of the reciprocal of the mean sensitivity of the thermistors determined from the calibration data, and
*c* is the specific heat capacity of water at the calorimeter operating temperature.

The product 1/2 (Δ*R/R*) $\left|{\bar{S}}^{-1}\right|$ is the temperature rise.

## 2. The Calorimeter

### 2.1 Design

**Fig. 1:**
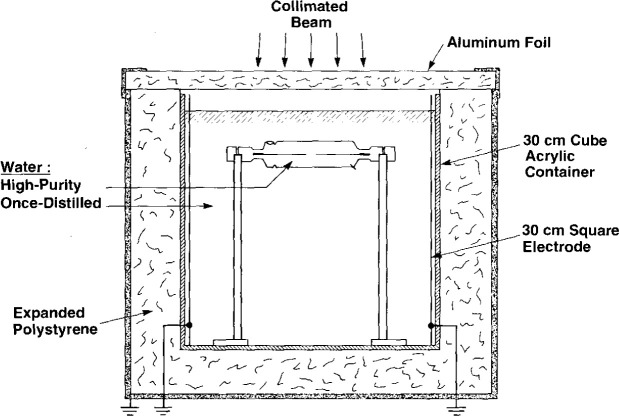
Essential features of the SW calorimeter for measuring absorbed dose or absorbed dose rate.

Figure 1 shows the general features of the sealed water (SW) calorimeter. “High-purity” water (HPW) is sealed within a thin-wall cylindrical glass container, 110 mm long and 33 mm in diameter. It has a dual purpose: (1) to enclose the sealed water, and (2) to act as a convective barrier. The assembly is immersed in a 30 cm cube acrylic container filled with once-distilled water (the same system used for the calorimeter described in Ref. [4]). The electrical resistivity of the water was about 0.4 MΩ·cm. A thermistor was mounted in the water to display its temperature to a resolution of 0.01 °C.

The name HPW is used in this paper merely to refer to the relatively cleaner stagnant water in the glass container. The water was prepared in a system consisting of a filter, deionizer, and an organic absorber, which gave the water an electrical resistivity of 30 MΩ·cm. The water was withdrawn and handled in glassware which had been cleaned and placed overnight in a furnace at 450 °C. The water in the glass container was saturated with the high-purity gases.

**Fig. 2:**
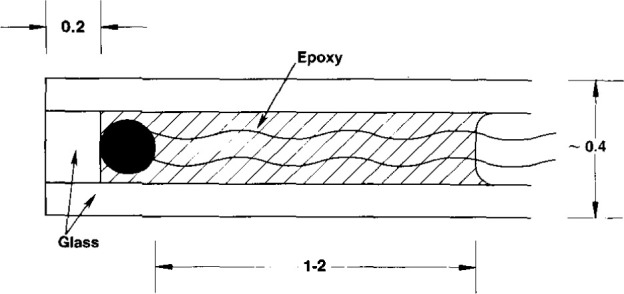
Schematic diagram showing the essential constructional details of the temperature probe consisting of an embedded thermistor near the end of a long thin capillary. The dimensions are in millimeters.

A square collimated ^60^Co beam (145 mm × 145 mm at the 50 percent dose points) produced a temperature rise, that was measured at a 5 cm depth from the water surface. The temperature probes consisted of the calibrated thermistors (0.25 mm in diameter) enclosed within and near the end of thin glass capillaries positioned along the axis of the tube. Figure 2 shows schematic constructional details of the sensor end of the probe. The distance between the thermistors can be varied. It was set at 9 mm within a uniform field.

**Fig. 3:**
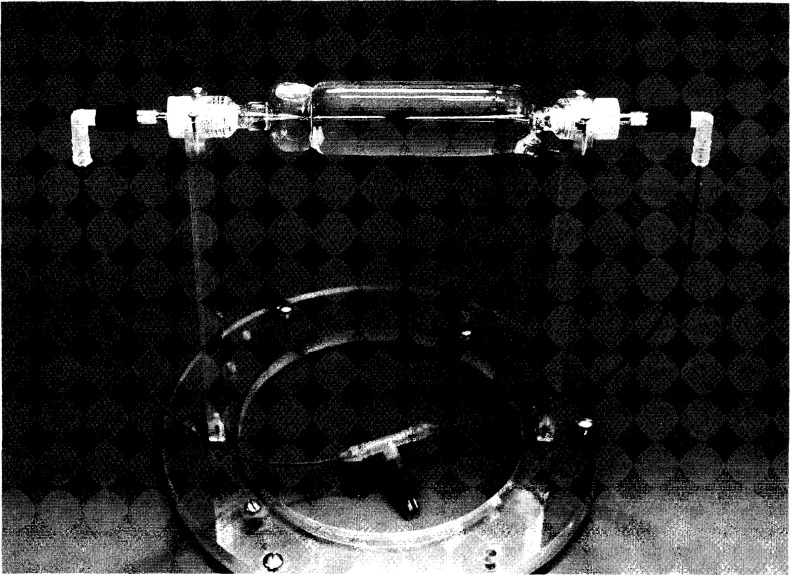
Photograph showing some details of the mounted thin-wall glass tube enclosing the HPW and temperature probes connected to external waterproof leads.

Figure 3 is a photograph showing some constructional details of the mounted detector assembly. The entire length of the glass structure is 175 mm. The outside diameter around the mid-plane is 33 mm, and the wall thickness varies from about 0.25 mm to 0.33 mm. The volume of the enclosed water is 90 cm^3^. Shown at the upper left within the tube (and indicated in Fig. 1) is a bubble of the saturating gas. Its purpose is to expand or contract, to prevent the tube from fracturing when the assembly is subject to large room temperature variations. The bubble is entrapped at that position by the circumferential depression in the tube, which prevents the bubble from migrating to the beam axis directly in front of the sensing thermistors. Shown at the lower right of the tube in Fig. 3 (and indicated in Fig. 1) is a platinum lead. It was in contact with the HPW and the once-distilled water, which was electrically grounded. The platinum lead was included as a precaution against possible charge buildup within the HPW, which could cause large disturbances in the small temperature signals that were to be measured. The large diameter portions (7 mm) of the temperature probes passed through snugly fitting holes in bushings of *low density* [(0.91–0.93) g/cm^3^] polyethylene, which is relatively elastic. High density [(0.94–0.97) g/cm^3^] polyethylene should *not* be used, because it is relatively rigid and stiff. The low density polyethylene, according to preliminary helium-leak tests, helped to make vacuum-tight seals between the polyethylene and glass when the bushings were screwed into position against glass apertures (shown below). This sealed the water from exposure to the atmosphere containing oxygen, which would otherwise be reabsorbed in the water to cause a heat defect. The total area of polyethylene in contact with the water was about 5 mm^2^. Small holes were drilled through the electrical sockets so that the air within the capillaries would vary with atmospheric pressure. If troublesome moisture condensed within the probes, it could be evacuated through the holes by placing the probes in a vacuum chamber.

### 2.2 Measurement and Drift Balancing Circuit

**Fig. 4:**
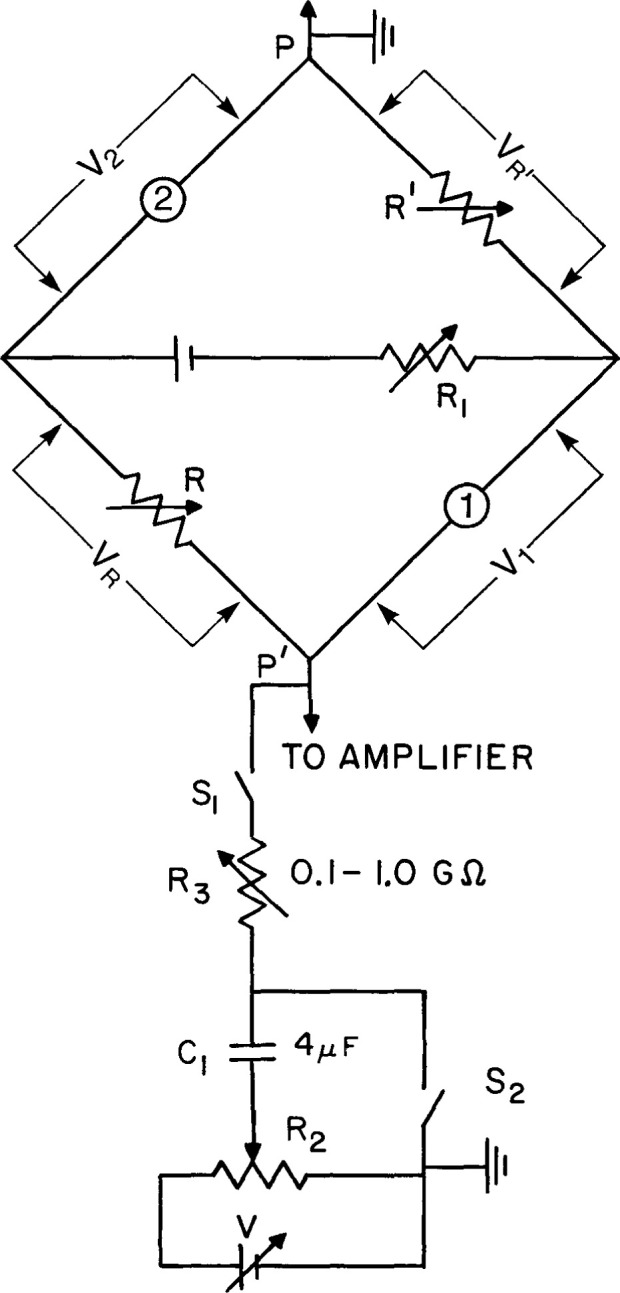
Wheatstone bridge circuit for detecting temperature changes and a resistance-capacitance circuit (temperature drift balancer) to counteract drifts in signal across PP′ as a result of temperature gradients in the vicinity of the thermistors.

The upper part of Fig. 4 is a Wheatstone bridge circuit containing thermistors 1 and 2 in the temperature probes. It is used for calibrating the thermistors and measuring the irradiation temperature response. The resistor R′ is preset so that R′ ≅ *r*_2_ (the resistance of thermistor 2), when V_2_ = V_R′_. When the bridge is balanced, the resistances of the four arms are approximately equal. These conditions will give near minimal corrections for changes in thermistor power during irradiation [4]. The resistor R_1_ is preset to give a desired average electrical power dissipation in the thermistors. Irradiation causes the bridge to be unbalanced, but is restored near the balance position by adjusting the bridge balancing resistor, R. A computer program analyzed the runs and calculated slight variable corrections (< 0.03 percent for powers up to 30 μW) for effects of slight changes in thermistor powers during a run. This correction is included in the value Δ*R/R* [in Eq. (1)], the fractional change in the bridge balancing arm.

The lower part of Fig. 4 is a resistance-capacitance (RC) circuit. Its function was described previously in detail [5–7]. Briefly, its purpose is to balance out small initial drifts in signal across PP′ caused by temperature gradients in the vicinity of the thermistors. This is done by moving R_2_ in such a direction as to produce a change in drift signal across PP′ from the RC circuit, which is opposite to that caused by the internal temperature gradients. When used for the measurements described in this paper, R_3_ was set on 0.5 GΩ to give a 33 min time constant, large in comparison to the radiation time. Irradiation times were varied and were nominally 1 min and 1.5 min.

### 2.3 Thermistor Calibration

The thermistor resistance value (*r*) at a given absolute temperature (*T*) is given by the well-known empirical expression:

$$r=r_{0}e^{\beta (1/T-1/T_{0})},$$ (2)

where *r*_0_ is the resistance at temperature *T*_0_. *β* is the “material constant,” which has the dimension of temperature (K).

The sensitivity of a thermistor (*S*) is defined as (1/*r*)(d*r*/d*T*), which gives

$$S=|-\beta /T^{2}|.$$ (3)

Equation (2) can be reduced to linear form:

y=βx+θ, (4)

where *y* = 1n *r, x* = 1/*T*, and *θ* = 1n *r*_0_ *− β/T*_0_, a constant. Least-squares fits of the data are applied to Eq. (4).

**Fig. 5:**
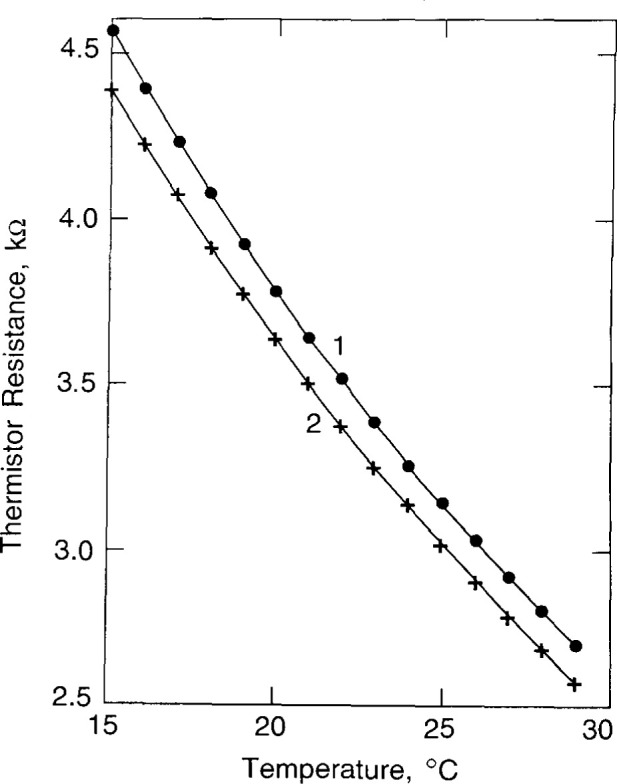
Calibration data of thermistor resistances as a function of temperature.

Calibration of a thermistor is the determination of *S*, which is its fractional change in resistance per degree change in temperature. The temperature was measured with a calibrated mercury thermometer (0.01 °C per division) and a quartz thermometer. The thermistors used had a resistance of about 3.3 kΩ at 22 °C with a negative coefficient of resistance of about 3.7%/K. The temperature probes were removed from the glass container and placed in the once-distilled water (Fig. 1) so that they would rapidly change with the water temperature which was varied at intervals of 1 °C from 15 °C to 29 °C. The temperature of the water was raised with four immersion heaters (total 100 W), and the water was circulated to attain uniform temperature. Then the water was allowed to become stagnant before measuring the thermistor resistances. Their resistances as a function of temperature are shown in Fig. 5. The bridge was balanced at each temperature. The thermistor resistances (*r*_1_ and *r*_2_) plus the external lead resistances, which were about 0.6 percent of *r*_1_ or *r*_2_, could be determined from the known resistances (*R* and *R′*) and the four measured potentials across the bridge arms as indicated in Fig. 4. The thermistors, however, were calibrated one at a time (for the sake of safety in handling), which required replacing a thermistor with a known resistance. This then gave two methods of determining a thermistor resistance, from (1) two measured potentials and a known resistance, and (2) three known resistances.

The effects of the external lead resistances decrease the sensitivity of the Wheatstone bridge arms containing the thermistors. This decreases the observed calorimetric signal. These are nullifying effects that lead to negligible measurement errors. The latter effect appears in the numerator of Eq. (1), and the former appears in the denominator. Therefore, small lead resistances can effectively be considered as an integral part of *r*, if no correction is applied to the observed signal. Otherwise, essentially the same correction factor must be applied to determine the true thermistor sensitivity and the calorimetric signal.

**Fig. 6:**
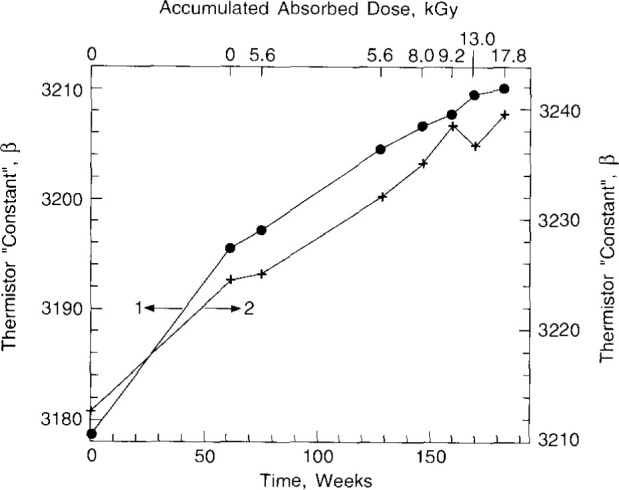
Thermistor “constants,” *β*, as a function of accumulated absorbed dose and time.

Figure 6 shows interesting calibration results measured eight times over a period of 184 weeks. They were first measured soon after their construction. The second calibration was done 62 weeks later (in preparation for irradiation measurements described below). Although they received no irradiation during this period, the *β* and *r* values had average increases of 0.45 percent and 3.5 percent, respectively. During the first phase of the experiment (5.6 kGy accumulated absorbed dose in N2-saturated water), they increased again by 0.03 percent and 0.34 percent, respectively. With no further increase in absorbed dose and 53 weeks later, the increases were 0.22 percent and 0.21 percent, respectively. Over the next 55 week period [(5.6–17.8) kGy accumulated absorbed dose in H_2_- and H_2_/O_2_-saturated water] the increases were 0.20 percent and 0.14 percent, respectively.

The changes appear not to have been caused by irradiation. This is consistent with other reported results of accumulated absorbed dose up to 620 kGy [8]. In another investigation [9] an accumulated absorbed dose of 4.3 MGy at a rate of 25 kGy/min from 3 MeV electrons produced a resistance change of only about 0.01 percent. A possible cause may have been a gradual release of strains within the thermistors as a result of their manufacture or handling their delicate leads during the fabrication of the temperature probes.

The above points out the precautions that must be taken to assure that the *β* values remain essentially constant during an extended experimental period. If they change significantly, it must be detected immediately and recalibrated. The method used was the observation that a change in *β* was accompanied by a change in *r.* The resistances were routinely measured before and after a daily set of measurements from the known potential and resistance values of the Wheatstone bridge arms as described above. The *β*-value changes between the dates of calibration were assumed to be linear for determining slight corrections to the date of absorbed dose measurements.

**Fig. 7:**
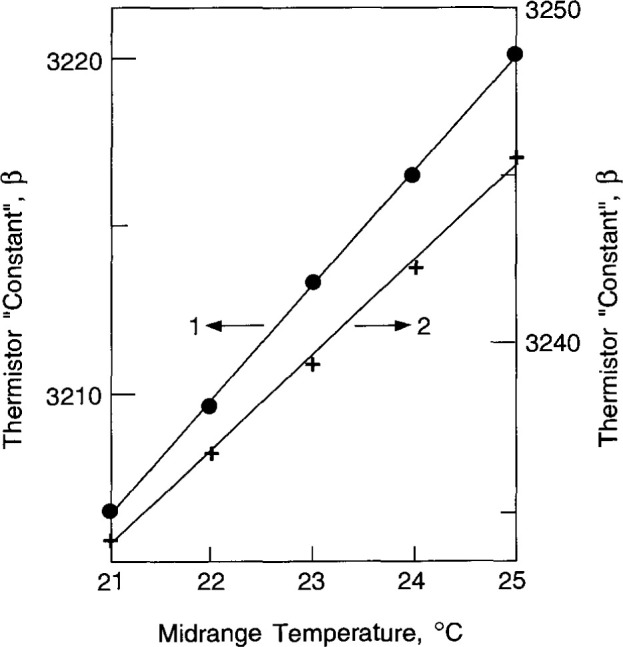
Thermistor “constants,” *β*, as a function of mid-range temperature calibration.

Table 1 lists a set of measured *β* values determined from different temperature ranges, which vary from three to six degrees above and below mid-temperature ranges from 21 °C to 25 °C. The average values are listed at each set of mid-range temperature and the experimental standard deviations of the mean, which average about 0.01 percent. The average values are plotted in Fig. 7. From all sets of measurements, the average increase in the *β* values was 0.11%/°C increase in mid-temperature calibration. The temperature of the thermistors was known during the measurements of absorbed dose (near 22 °C or 23 °C). Small corrections were made to the *β* values because the temperatures differed from those nominal values.

## 3. Heat Flow Calculations

Ideally, the temperature rise at a point in water would be made with a massless sensor. But this is not realizable in practice. Thermistors are made of metallic oxides, which are covered with glass beads in the manufacturing process. The thermistor and its bare leads are then embedded in a thin glass capillary to electrically insulate it from the grounded water. Temperature rises, therefore, are measured primarily in glass.

The immediate rate of temperature rise of the irradiated glass, over a wide range of photon and electron energies, is about four times that of the surrounding water. Reference [10] gives useful information on the temperature rises of various materials in the form of a foil, wire, or small sphere, such as a thermistor.

Irradiation causes excess heat to be generated in the temperature probe. The amount of this heat is proportional to the mass of glass, which therefore must be made as small as possible. If measurements are to be accurate, the excess heat must be rapidly conducted away during irradiation and reduced to negligible amounts within a few seconds after irradiation. This is the start of the calorimetric afterdrift, which is used in the analysis for determining the temperature rise. Some excess heat will remain during this period. Heat-flow calculations must be made to determine if the non-water materials can be made small enough, so that the effects of the remaining excess heat do not significantly affect the accuracy of measurements.

With reference to Fig. 1, there are three sources of excess heat if the assumption is made that the HPW within the glass container has a zero heat defect with a relative absorbed dose rate of 1. These are convenient assumptions for the sake of carrying out the calculations described below. The sources of excess heat are the (1) once-distilled water, (2) thin glass wall, and (3) glass capillaries. Calculations were performed by use of the well-known Schmidt numerical method described in many publications as, for example, in Ref. [5]. The water was imagined to consist of concentric cylindrical shells (0.25 mm thick) with a common axis along the axis of the temperature probes.

### 3.1 Effect of Once-Distilled Water

**Fig. 8:**
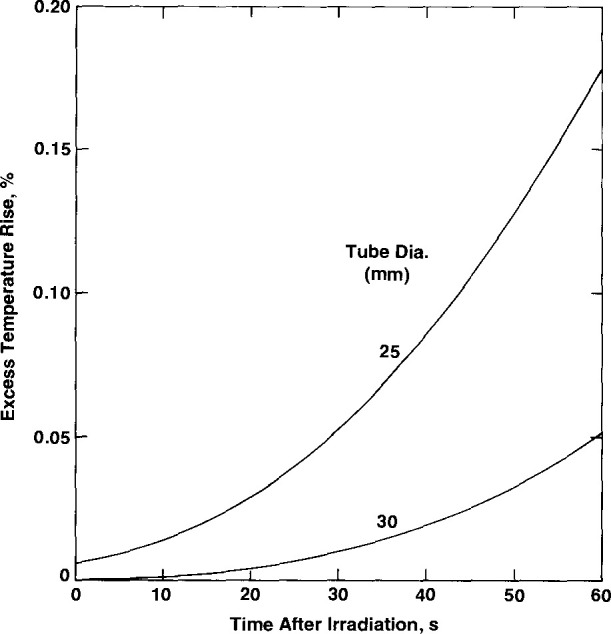
Excess thermistor temperature rise caused by heat conduction from once-distilled water outside a tube (25 mm and 30 mm in diameter) as a function of time after a 60 s irradiation run.

The once-distilled water was found to have an exothermic effect of 3.5 percent [4]. Therefore, the relative absorbed dose rate in this water is 1.035. This will cause excess heat to be conducted cylindrically toward the thermistors. Figure 8 shows the excess temperature rise (in percent) of the thermistors as a function of time after a 60 s irradiation run. The effect is greatly reduced when the tube diameter is increased from 25 mm to 30 mm.

### 3.2 Effects of Glass Wall

The glass wall is an effective barrier against convection, which can occur relatively easily in the large external volume of water at room temperature [11,12]. Its diameter (*d*) must not be too small, because significant excess heat from it may arrive at the thermistors before the irradiation run is completed. It must not be too large, because conditions will eventually occur when the onset of convection will take place (when the Rayleigh number ⩾ 1000) [4,13,14]. Results from the previous and following numerical calculations were valuable in deciding on an appropriate diameter.

**Fig. 9:**
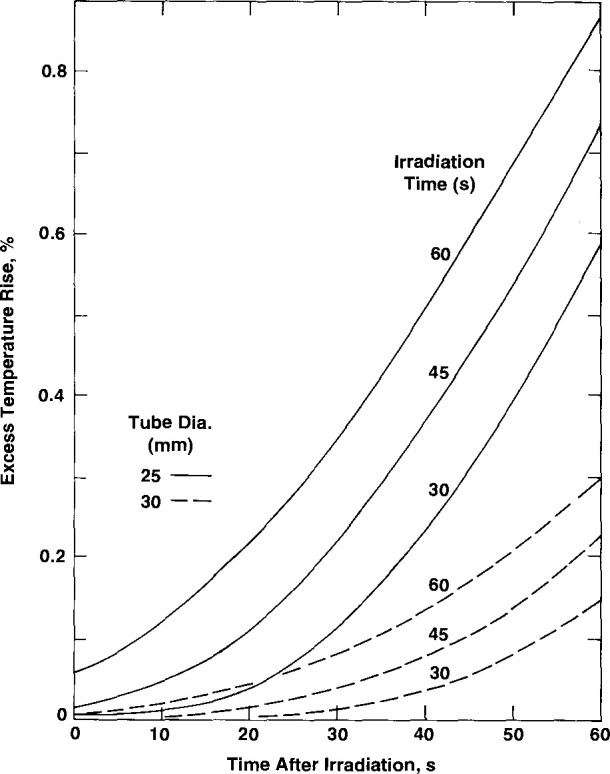
Excess thermistor temperature rise caused by heat conduction from a 0.25 mm thick glass wall tube (25 mm and 30 mm in diameter) as a function of time after irradiation.

A uniform glass wall thickness of 0.25 mm was assumed for the calculations. A further simplification was made to facilitate the calculations. Considering the mass energy-absorption coefficients of glass and water, their densities, and specific heat capacities, it can be shown that the glass can be imagined as being replaced with water where the relative absorbed dose rate is about 2.4 for the case of ^60^Co irradiation. Excess heat will be conducted cylindrically away and toward the thermistors. Figure 9 gives the excess temperature rise (in percent) of the thermistors as a function of time after a 30 s, 45 s, and 60 s irradiation run. The results show that the tube diameter should be 30 mm or somewhat greater. The constructed tube shown in Fig. 3 has an outside diameter of 33 mm in the region of the thermistors. It appears that the diameter should not be larger than 35 mm. At that diameter the calculated excess temperature rise is 0.09 percent, at 60 s after a 60 s irradiation run. In practice, extrapolation of the final drift to the mid-run will *tend* to correct for the variable increase of the small amount of excess heat sensed by the thermistors; or the calculated curves shown in Figs. 8 and 9 could be included in a computer program and subtracted from a measured post-irradiation drift.

### 3.3 Maximum Effect of Temperature Probe

**Fig. 10:**
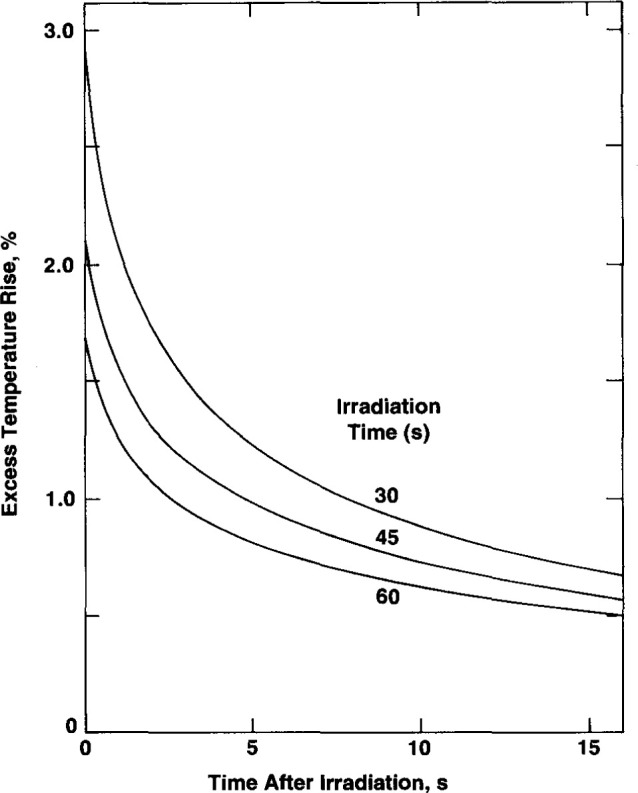
Excess temperature rise at the axis of a long solid glass rod (0.25 mm radius) immersed in water as a function of time after irradiation.

The basic detailed construction of the temperature probe is illustrated in Fig. 2. The thermistor is located near the end of the probe and embedded in epoxy. The excess heat is conducted cylindrically and spherically away from the region near the end of the probe; and while this is happening, heat is conducted axially along the capillary toward the end of the probe. The combined effect of this geometry and of the densities and thermal conductivities of the glass and epoxy is a complex situation that is under detailed study at the time of this writing. However, a maximum effect (greater by a factor of about 7) was determined by calculating the excess temperature rise at the center of a long solid glass rod 0.5 mm in diameter. There is then only cylindrical flow of heat. This is easily solved by the numerical method mentioned above. The results are shown in Fig. 10. Note that if significant excess heat remains at the end of the irradiation period, there will be a noticeable sharp drop in signal within 5 s after irradiation. During actual measurements (shown below) this was too small to be observable.

### 3.4 Orientation of Probes

To avoid concerns and possible errors in measurement, it appears that positioning the axes of the temperature probes perpendicular to the beam (as shown in Fig. 1) is better than positioning a pair of probes parallel to the beam [11]. The concerns and errors would depend on the absorbed dose (temperature) profile along the probe, its geometry, and the thermal diffusivity of the pyrex glass probe which is about four times that of water. Measurements are commonly made at the peak of the absorbed dose curve, where the radial temperature gradients are usually much smaller than the axial gradients. This might cause a component of heat to flow along the temperature probes (parallel to the beam) significantly different from that through water.

## 4. Probe Fabrication

### 4.1 Capillary Formation

**Fig. 11:**
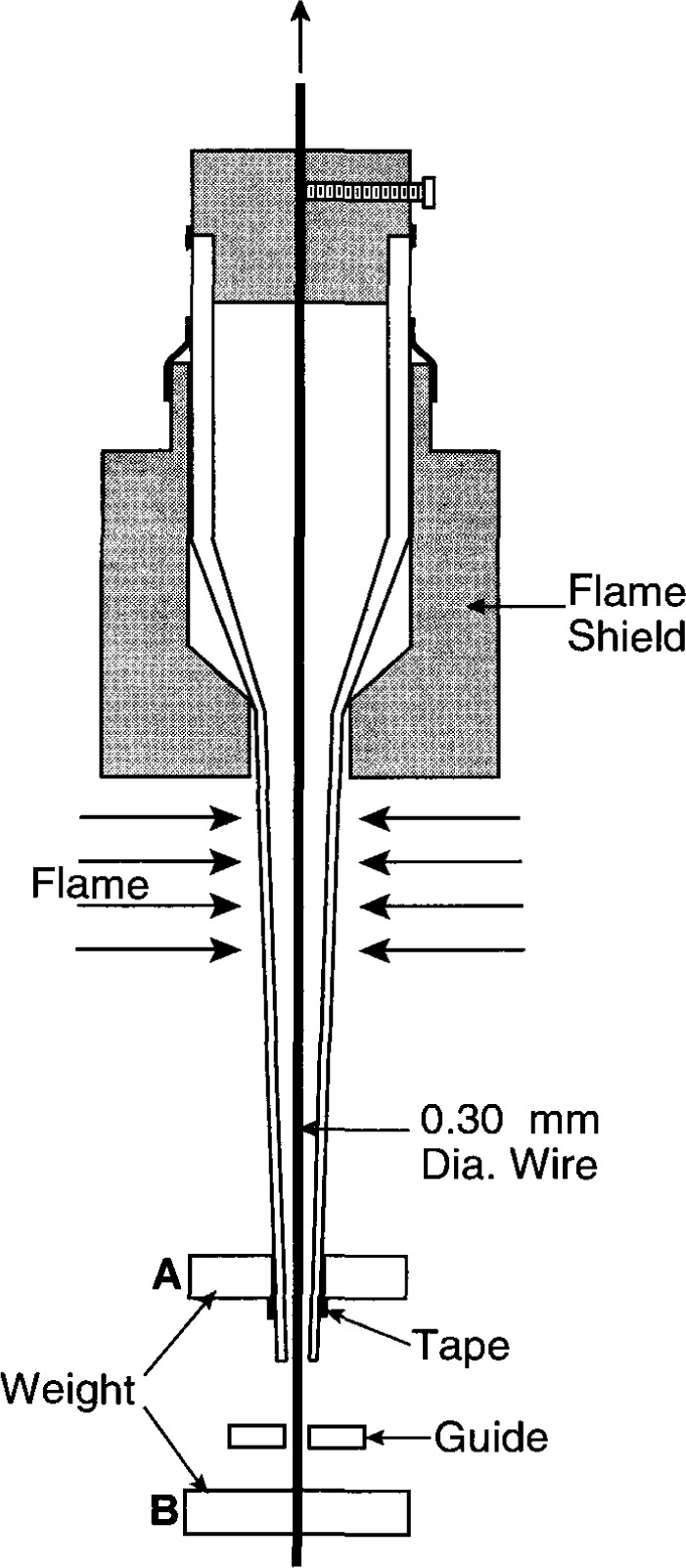
Illustrative method of reducing the diameter and wall thickness of a pipet to desirably small dimensions.

The temperature sensing probes were fabricated from commercially available pyrex pipets. Figure 11 illustrates the first step. Weight A (about 3 g) was attached near the lower end, which had nominal inside and outside diameters of 1 mm and 1.5 mm, respectively. These dimensions are too large. The inside diameter was to be formed by a high-melting-point wire, 0.30 mm in diameter. This would barely allow the thermistor to be inserted into the completed capillary. Weight B (about 300 g) was attached to the lower end of the wire, to keep it straight. The guide hole held the assembly in a vertical position. The flame shield offered some protection from distortion of the enclosed glass surface, which was to be part of the sealing surface for enclosing the HPW. Two torches were used to direct flames along opposite sides of the pipet. When the glass softened, weight A caused the pipet to stretch and collapse around the wire. The formed capillary and enclosed wire were cut. This shattered a small section of the capillary and blunted the end of the wire, which was filed to a point to permit its removal. It was successfully removed, roughly about 75 percent of the time without breaking the thin-wall capillary. The glass did not fuse to the wire, because it was pre-blackened over a candle flame. It was also pre-strained to remove its curvature, which would otherwise cause a force against the thin capillary and possibly cause it to break when weight B was removed. Although the capillary diameters over a distance of at least 1 cm from the end ranged only 0.38 mm to 0.44 mm (measured with a graduated microscope disk), they were sufficiently sturdy. Capillaries with larger diameters were rejected.

### 4.2 Grinding

**Fig. 12:**
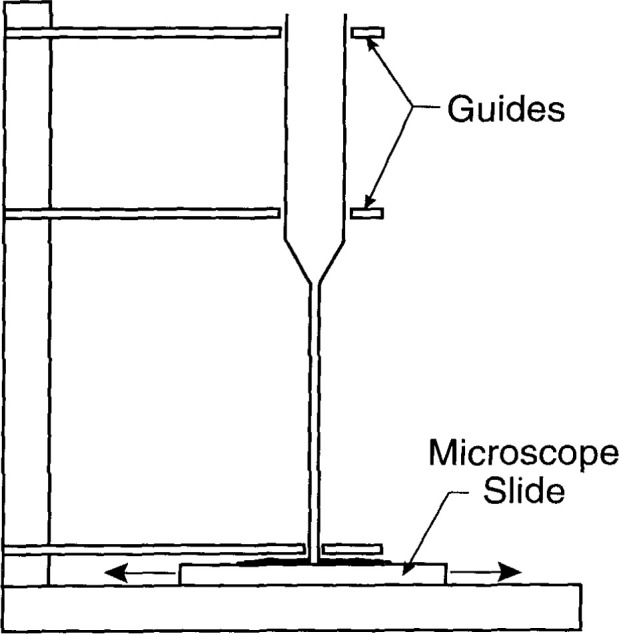
Illustrative method of grinding the end of a capillary.

Figure 12 shows the method used for grinding the end of the capillary. The shattered end rested on a microscope slide containing a mixture of fine grinding powder and water. After the shattered end was ground off, the unit was heated in a furnace at 565 °C for several minutes to remove possible strains in the glass.

### 4.3 Aligning

**Fig. 13:**
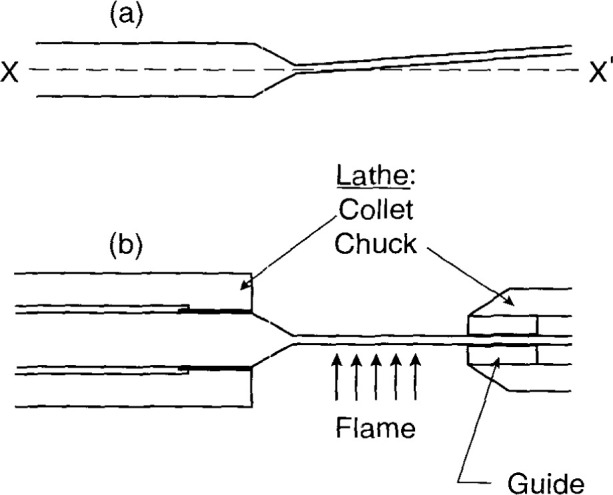
Illustrative method of making the sensor end of the capillary axis coincide with the larger tube axis.

Figure 13(a) indicates that the XX′ axis of the probe large diameter (7 mm) did not necessarily coincide with the axis of the small capillary. This happened even though (as illustrated in Fig. 11) the assembly was held in a vertical position. The angle of offset was random. A significant offset would cause the thermistor to be unnecessarily too close to the glass wall when it was screwed into the assembly as shown in Fig. 3. This would cause the excess heat from the wall to arrive sooner at the thermistor. The most important part of the offset (toward the thermistor) was removed by the method illustrated in Fig. 13(b). The large diameter end was held in a lathe collet. The long thin capillary was flexible enough so that part of it was made to pass through a hole in a guide held with the lathe chuck. The lathe was turned at a slow speed (~ 25 rpm) while a flame was directed on the capillary as shown. When the glass softened, the strain along the capillary was relieved and then the flame was removed. This caused the thermistor to be aligned close to the XX′ axis.

### 4.4 Encapsulation

#### 4.4.1 Thermal Coupling

**Fig. 14:**
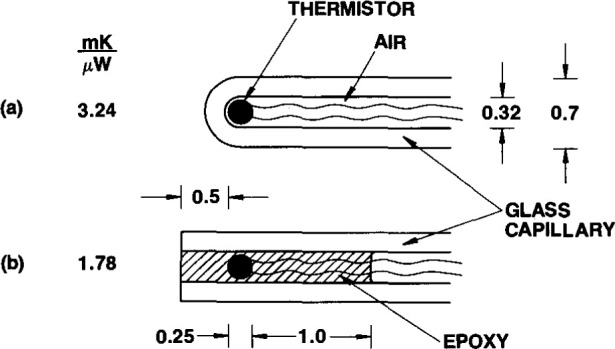
Results of measurements of the temperature rise in a thermistor per unit electrical power dissipation when it was surrounded directly by: (a) air; (b) epoxy. The dimensions are in millimeters.

A desirable characteristic of a temperature probe is to have good thermal coupling between the thermistor and its surroundings. Figures 14(a) and 14(b) show the results of preliminary work to determine the method to be used and its effectiveness in enclosing a thermistor [15]. When enclosed and surrounded by air as indicated in Fig. 14(a), the immersed probe had a temperature rise of 3.24 mK/μW of electrical power dissipation. The end of the long capillary was then ground off and the thermistor was embedded as shown in Fig. 14(b). Its temperature rise was then 1.78 mK/μW. This lower value is an improvement and is attributed mainly to the higher thermal conductivity of the epoxy, about 30 times that of air.

Further preliminary work removed possible troublesome features shown in Fig. 14(b), which indicates that the surface of the epoxy would be in contact with the HPW that would absorb organic impurities. Furthermore, prolonged immersion would cause the electrically grounded water to be absorbed in the epoxy and be in contact with the thermistor and its leads, which would cause large erratic signals. This possible source of trouble was eliminated by closing the capillary end with glass as illustrated in Fig. 2.

#### 4.4.2 Lead Insulation

**Fig. 15:**
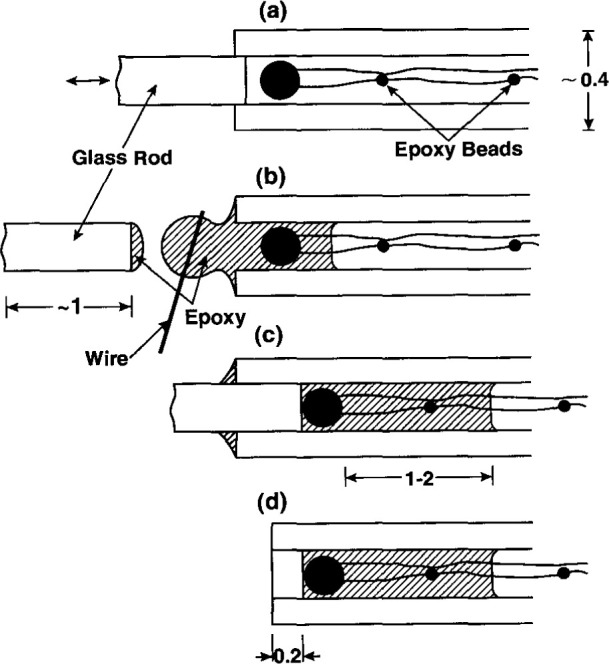
Illustrative procedure for embedding and enclosing a thermistor and ensuring that its bare leads would not be electrically shorted. The dimensions are in millimeters.

Figure 15 illustrates the procedure for embedding, enclosing, and ensuring that its bare Pt-Ir leads (25 μm in diameter and ~ 1 cm long) would not be electrically shorted. The leads were soldered to 25 μm diameter copper wires coated with polyurethane. To prevent electrical shorts, particularly in the congested region of the soldered joints, observations through a microscope revealed the necessary locations on which to apply beads of fast drying epoxy. Then the leads and thermistor were withdrawn and the epoxy beads were applied. Several cycles like this had to be made before observations revealed that the epoxy beads separated the bare portions of the wires.

#### 4.4.3 Embedment

Figure 15(b) shows the application of a viscous slow-drying epoxy at the opening of the capillary. A thin wire was used to transport several applications of the epoxy into position with the aid of a microscope. The epoxy was drawn into the capillary by the surface tension caused by those materials. The amount of epoxy applied was such that its final length behind the thermistor was 1 mm to 2 mm, as indicated in Fig. 15(c). This provided increased thermal conductivity from the heated wires close to the thermistor with only a slight addition of a non-water material near the thermistor.

#### 4.4.4 End Closure

The glass rod (~ 1 mm long) was attached to an apparatus containing three micrometer movements that were necessary for maneuvering the glass rod into the capillary opening. The glass rod was made slightly tapered, and its ground right surface had a diameter only about 10 μm to 15 μm smaller than the opening of the capillary. The inserted rod pushed back the thermistor, making a direct contact. The taper of the rod was such that the glass rod was also in firm contact with the capillary. The capillary end shown in Fig. 15(c) was ground down to the 0.2 mm dimension shown in Fig. 15(d). Placement of the thermistor close to the capillary end causes it to be in the “cool” region of the excess heat, because of the added and strongest heat conductivity (spherical) from the capillary end.

**Fig. 16:**
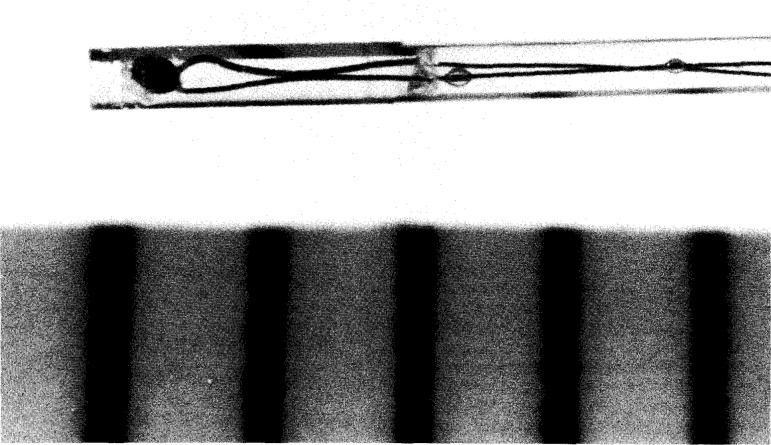
Photograph of the constructed temperature probe. Reflections make it appear that there is an abrupt change in capillary diameter near the right end to the enclosed epoxy. The scale is in millimeters.

**Fig. 17:**
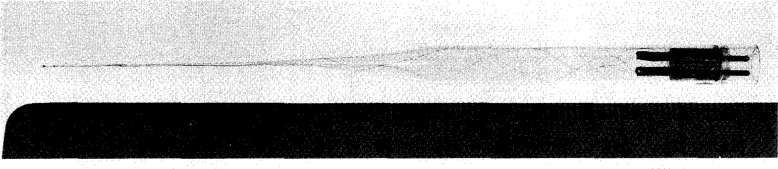
Photograph of the temperature probe assembly.

Figure 16 is a photograph of the sensor end of the completed temperature probe. The scale markings are in millimeters. Reflections and refractions make it appear that there is an abrupt change in capillary diameter at a position near the right end of the enclosed epoxy. Figure 17 shows the entire probe assembly. The electrical plug was epoxied into position. Two holes were drilled parallel to its axis in order to not entrap the air within the capillary. Six probes were constructed. When immersed in water, their temperature rises ranged from (1.34–1.56) mK per μW of electrical power dissipation.

**Fig. 18:**
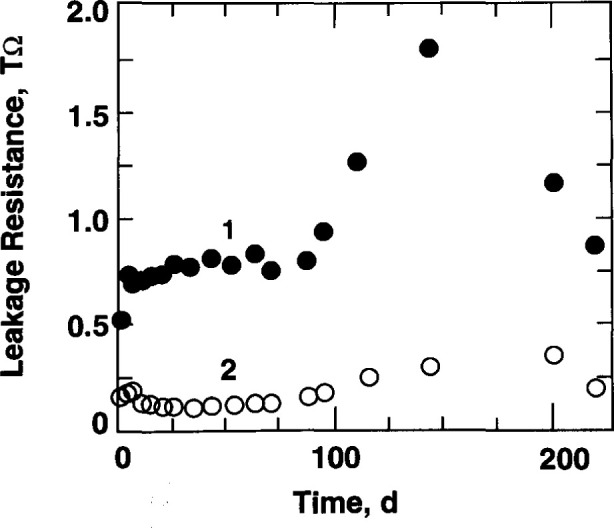
Measurements of leakage resistance between thermistors 1 and 2 in the temperature probes and the surrounding water.

The setup shown in Fig. 1 was used to measure the leakage resistances between the thermistors 1 and 2 in the temperature probes and the surrounding water, which remained in the glass container for 221 d. The results are shown in Fig. 18. The resistances were always high and stable over long periods compared to the irradiation time. A calculation based on an assumed maximum and rapid short time change showed that the resultant contribution to the noise level would be lower by a factor of about 500 compared to the noise observed during the measurements.

## 5. Heat Defect

The heat defect, *k*_hd_, is defined by

$$k_{\text{hd}}=(E_{a}-E_{h})/E_{a},$$ (5)

where *E*_a_ is the energy imparted to a material and *E*_h_ is the energy released as heat. The reaction is endothermic when *E*_a_ > *E*_h_ and exothermic when *E*_h_ > *E*_a_.

The heat defect was perhaps the greatest obstacle to initiating the investigation of the water calorimeter. The initial design consisted of once-distilled water open to air and in contact with plastic materials [3,4]. Exothermic results reported by investigators ranged from 1 percent to 5 percent. It is difficult to determine how much of this can be attributed to differences in water quality. Part of it can be attributed to real errors in the comparative method of ionization chamber measurements and the use of protocols to determine absorbed dose.

Significant progress has since been made in carrying out the above mentioned second and relatively longer phase (2) in the development of the water calorimeter [4]: theoretical and experimental investigations aimed at giving more assurance concerning the heat defect.

Therefore, the new generation of calorimeters are to be relatively clean, to improve the water quality. The water is to be contained in clean glass containers, and sealed from the atmosphere [16,17]. It is also to be prepared in such a way as to cause an essentially zero heat defect [16–20], or to determine if a particular type of heat defect could be reproduced and accurately corrected [20]. These studies in the present experiment required saturating the HPW with high purity gases. The stated commercial minimum purities of the N_2_, H_2_, and O_2_ gases used were 99.9995 percent, 99.9995 percent, and 99.997 percent, respectively.

**Fig. 19:**
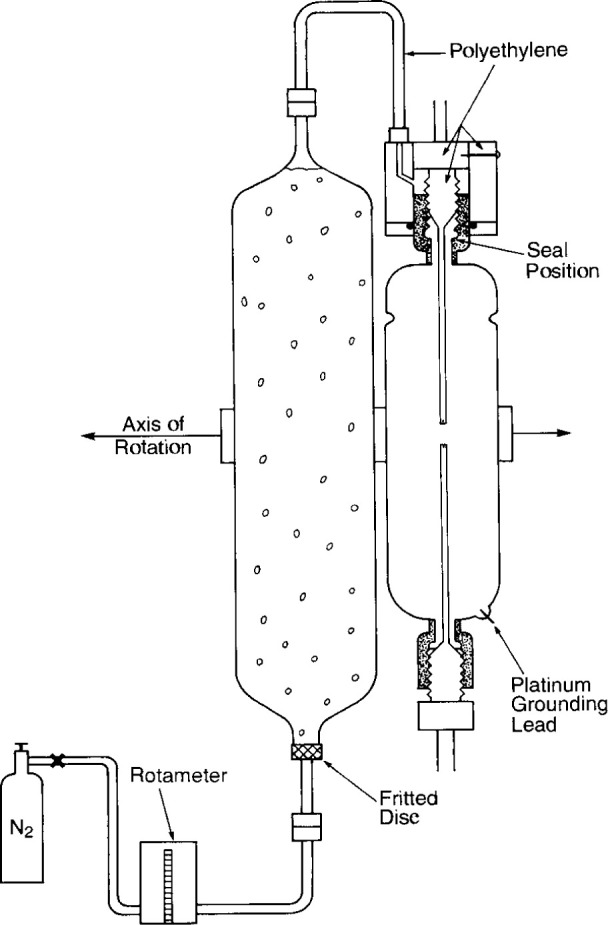
Illustrative method of gas saturating the HPW, which was then transferred to and sealed in the shown thin-wall glass tube. The drawing is not to scale.

Figure 19 illustrates the method of using the high-purity N_2_ (<0.2 parts per million O_2_) to replace absorbed gases in the HPW, which is then transferred to the glass detector assembly. A vacuum pump was initially used to evacuate possible trapped air pockets within the gas gauges and valves (not shown). The detector assembly was initially flooded with N_2_. Polyethylene tubing (3 mm inside diameter) connected the components shown. A rotameter indicated that the N_2_ flow rate was about 30 cm^3^/min. The gas passed through a fritted disk at the bottom of a glass column containing the HPW. The column had a volume six times that of the glass detector assembly. A vigorous flow of many tiny bubbles of N_2_ ascended through the water. The gas easily passed through the assembly, because the polyethylene bushings had been partly unscrewed to make the threads loosely fitting. The flow rate continued for 40 min. Then the column and detector assembly were rotated about 1/2 turn around the horizontal axis, while the gas pressure continued. The water ascended, entrapping a bubble of N_2_ in the region of the platinum grounding lead. When almost all the water was gone from the column, the bushings were screwed in to make the seals against the circular glass openings.

## 6. Measurement Procedure

The following describes (1) tests which show that the absorbed dose measurements were made in the absence of convection, (2) a series of radiation runs resulting in increasing temperature gradients and temperature drifts, and (3) a method of rapid restoration to equilibrium.

**Fig. 20:**
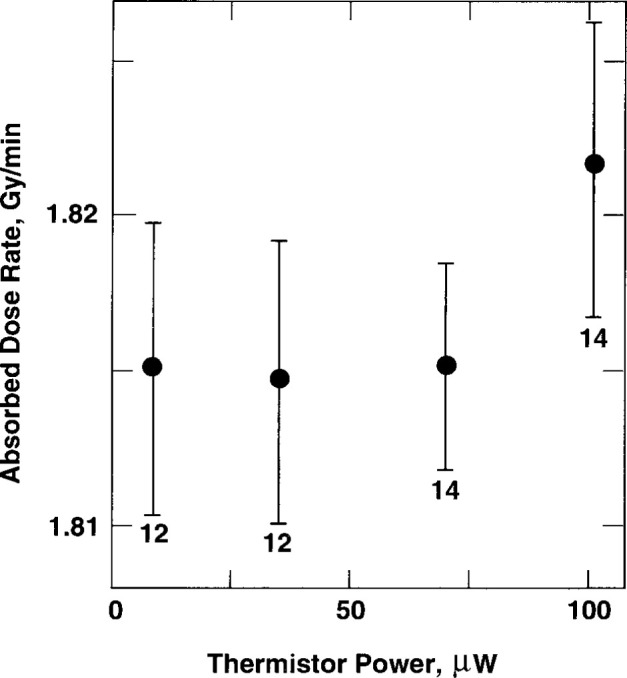
Absorbed dose rate measurements as a function of thermistor power.

Figure 20 shows results of absorbed dose rate measurements as a function of thermistor power. The uncertainties are the experimental standard deviations of the mean for the number of indicated measurements. Reference [21] describes convective velocity effects on an electrically insulated thermistor (0.25 mm in diameter) in water, free of convective barriers. It was shown that convection began around the heated thermistor when the electrical power was about 50 μW. Its measured temperature rise per μW of power was 1.41 mK. Therefore, convection began when its temperature rise was about 70 mK. The threshold of convection also will depend on thermistor size [12] and details of construction. The two temperature probes used in the present investigation had measured temperature rises of 1.33 mK and 1.53 mK per μW of power. Therefore, convection would begin when the average thermistor power would be raised to about 50 μW in water free of convective barriers. But the probes were mounted within the glass tube (Fig. 3) where its wall was a convective barrier. It is uncertain if this barrier raises the electrical power at which convection begins. Therefore, in the operational procedure it is safer to assume that the wall has no effect, for this case, and to use power levels significantly below 50 μW. Although Fig. 20 shows that there is no significant difference in the measurements up to 100 μW, the maximum power used was 30 μW. This is significantly below the threshold of 50 μW at which convection begins around a thermistor as described above. Many of the absorbed dose measurements were made at 9 μW. The range of powers from 9 μW to 100 μW caused the thermistors to rise to average equilibrium temperatures of 13 mK to 143 mK above the background water temperatures, while the temperature rise during irradiation caused them to rise an additional temperature of only about 0.5 mK (shown below). Therefore, slight disturbances of the equilibrium temperatures caused by irradiation produced convection (if present) would have resulted in significant changes in the measurements as a function of thermistor power.

**Fig. 21:**
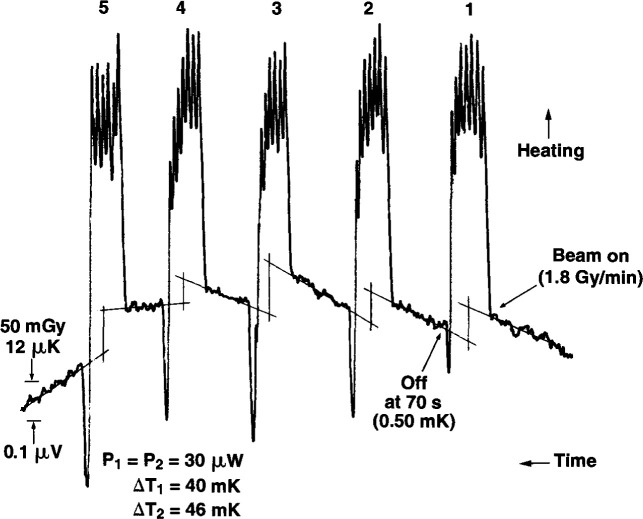
Typical consecutive runs showing measurement parameters and changes in temperature drifts as a result of heat conduction caused by collimated beam irradiation. Time increases from right to left.

Figure 21 is a typical series of radiation runs. Time increases from right to left. A small initial heating drift was allowed. This permitted five runs to be made in a series of runs where the drifts were small. The ^60^Co beam produced a dose rate of about 1.8 Gy/min. The spikes shown are caused by the heating and manual adjustments in the bridge balancing resistor, R. The duration of the runs was varied around 70 s to produce a temperature rise of only about 0.5 mK. The runs were analyzed in the usual way by extrapolating the initial and final drift to the mid-run, and determining the offset by calibrating the chart deflection from a known resistance change in R. A computer calculated the resistance value of R that would rebalance the bridge.

The first three temperature drifts are essentially unchanged. The fourth drift shows a small but significant change. The fifth drift shows a slight cooling, followed by the sixth drift, which shows significant cooling. This typical behavior is a result of outward conduction of heat from the penumbra of the collimated square beam, approximately 145 mm at the 50 percent dose level. Initially, the thermistors did not sense the outward flow of heat. Each thermistor had an electrical power dissipation of 30 μW, which caused an equilibrium temperature rise of 40 mK in thermistor 1 and 46 mK in thermistor 2. Superimposed on these temperature rises were the 0.5 mK temperature rises as a result of beam irradiation. The results shown in Fig. 20 and the predictable behavior of the successive drifts shown in Fig. 21 are indications that the glass wall is an effective convective barrier against external convection and that it is unnecessary to operate the calorimeter at 4°C [11].

The drift balancer circuit shown in Fig. 4 can balance out large drifts. But generally, large temperature gradients within the calorimeter should be removed and equilibrium restored. Equilibrium was restored after eliminating the temperature gradients by circulating the once-distilled water shown in Fig. 1 (in most cases this circulating procedure was done after only two consecutive runs). The water was slightly heated with the immersion heaters and circulated with an aquarium pump, which forced air to rise along the four vertical corners of the acrylic container. After several minutes the pump was turned off and the water gradually became stagnant. The drift continued because of a temperature difference between the circulated water and that within the glass container. The heating or cooling drift gradually became smaller as conduction continued, and within approximately 20 min (depending of the initial temperature difference) the drifts were small enough to continue another series of runs. Approximately 40 runs could be made in a period of 5.5 h.

## 7. Results of Absorbed Dose Measurements

### 7.1 N_2_-Saturated Results

All measurements refer to a linear depth of 5 cm and were normalized to a particular date to correct for ^60^Co decay. Slight corrections normalized the measurements to a calorimeter operating temperature of 22.0 °C, where the density of water is 0.9978 g/cm^3^. The specific heat capacity of water at this temperature was taken to be 4.1808 J/(g·°C) [22], which is assumed to be the same for air-saturated and air-free water [23]. A small estimated correction (mentioned below) was made for the effect of the thin glass wall.

**Fig. 22:**
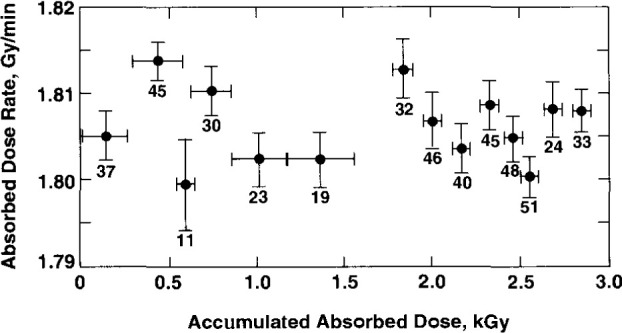
Results of 14 d of measurements of absorbed dose rate vs accumulated absorbed dose in the first N_2_-saturated water filling. The numbers indicate the number of measurements made on a day. There were 58 elapsed days from the first to the last day of measurement.

Figure 22 shows the number of daily measurements with the first water filling irradiated with an accumulated absorbed dose of 2.9 kGy. Measurements were made on 14 d over a time period of 58 d. The vertical and horizontal bars indicate, respectively, the standard deviations of the mean and the accumulated absorbed dose. It is assumed that the variations shown are statistical. On the first day there was an initial variation, which was not noticeable on subsequent days. The first measurement was about 6.5 percent higher than those after the 7th run (accumulated absorbed dose of about 20 Gy), where the measurements thereafter appeared to show statistical variations. This was the same general type of behavior observed on the first day of measurement after other water fillings, described below. The initial measurements showed exothermicity, which rapidly vanished and were ignored, as discussed below.

**Fig. 23:**
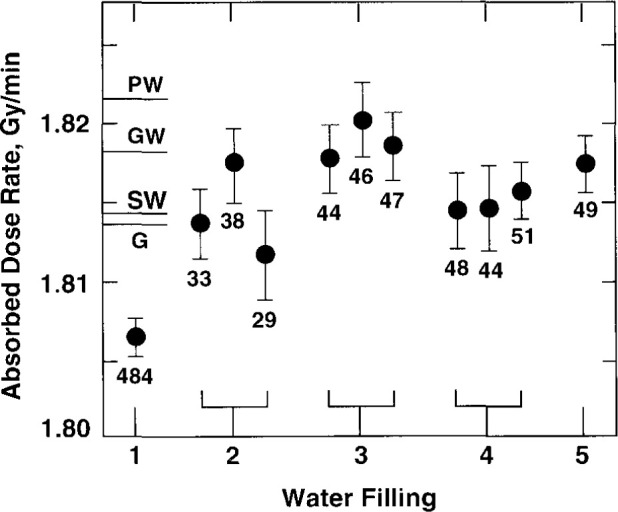
Results with five different N_2_-saturated water fillings. The average result is indicated (SW) and compared with results determined with other calorimeters: PW (polystyrene-water), GW (graphite-water), and G (graphite).

On some days, measurements began with a group of measurements followed by continuous unrecorded irradiation from 30 min to 3 h and then repeating the measurement cycle. On other days, the first two steps were reversed. On a few days only measurements were made. The numbers shown in Fig. 22 indicate a total of 484 measurements. The average of the 14 daily average values is shown as the first point in Fig. 23. The experimental standard deviation of the mean of the 14 average values is 0.07 percent.

The water in the glass container was discarded and filled four other times. Figure 23 shows the average results of the daily measurements. Initial variations were again observed on the first day of measurement, after the second, third, and fourth filling. After the second filling, the first measurement was about 5.5 percent higher than those after the 12th run (accumulated absorbed dose of about 34 Gy). After the third filling, the first measurement was about 7 percent higher than those after the sixth run (accumulated absorbed dose of about 10 Gy). After the fourth filling, the first measurement was about 3.5 percent higher than those after the second run (accumulated absorbed dose of about 5 Gy). After the fifth filling, the calorimeter was pre-irradiated for 45 min (accumulated absorbed dose of 70 Gy). Subsequent measurements showed only statistical variations.

For the second, third, and fourth fillings, Fig. 23 shows three average results from measurements made on three days with each of those fillings. The average of each of those three values (for a particular filling) was assigned as a single value for that filling. The average of the five values is 1.814 Gy/min, which is indicated as SW. The standard deviation of the five averages is 0.27 percent, which indicates the reproducibility of measurements with the water fillings. This is in good agreement with the 0.25 percent standard deviation determined by Schulz et al. [17], who used a similar water purifying system and saturated the water with high-purity nitrogen.

**Fig. 24:**
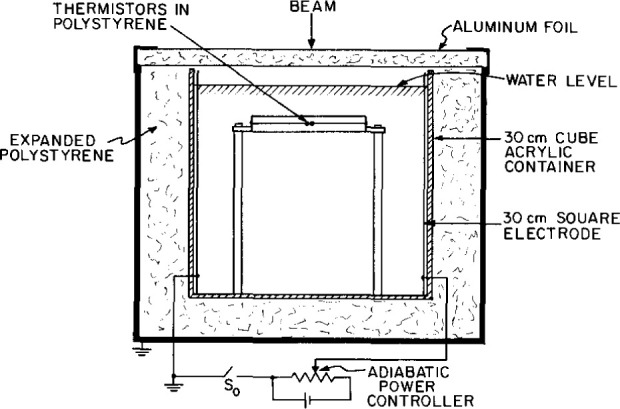
Essential features of the absorbed dose polystyrene-water calorimeter. The “adiabatic” power controller permits a potential to be applied across the electrodes so that in the vicinity of the 10 mm thick polystyrene disks the rate of temperature rise of the water, as a result of electrical power and beam irradiation, is as nearly as possible the same as that of the disks.

**Fig. 25:**
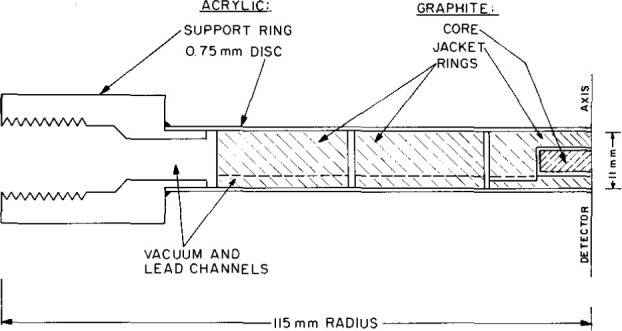
Essential features of the detector assembly for the absorbed dose graphite-water (GW) calorimeter.

**Fig. 26:**
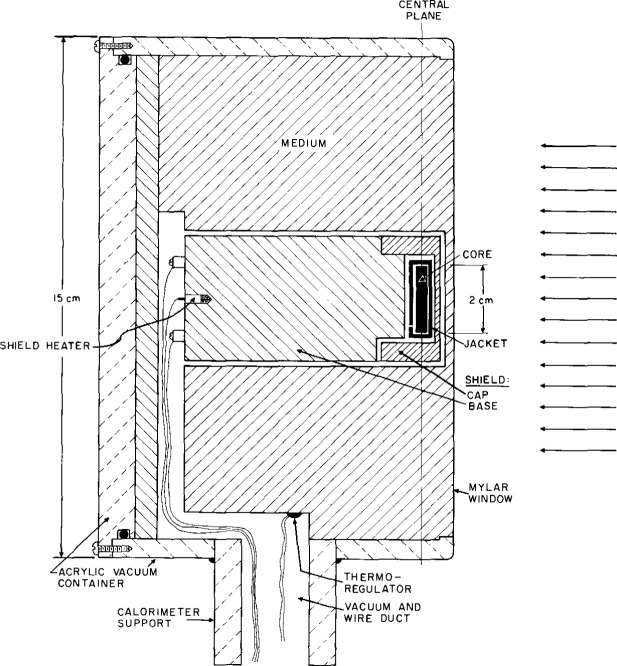
Side-view cross section of the graphite (G) absorbed dose calorimeter.

Figure 23 shows results of comparable absorbed dose determinations converted to water as measured with a polystyrene-water (PW) calorimeter [7] shown in Fig. 24, a graphite-water (GW) calorimeter where the schematic detector details are shown in Fig. 25, and a graphite (G) calorimeter [24] shown in Fig. 26. Measurements with the PW and GW calorimeters were converted to absorbed dose to water by use of the mass energy-absorption coefficients. Conversion of the G calorimeter measurements is described by Pruitt et al. [25]. The estimated combined standard uncertainties of the measurements with the PW, GW, and G calorimeters are 1.1 percent, 0.7 percent, and 0.6 percent, respectively.

The PW result is based on the specific heat capacity of polystyrene, which was calculated from an empirical equation [26]:

$$c={\left(104.15\right)}^{-1}\left(7.7551\cdot 10^{5}T^{-2}+0.53447T-41.58\right)J/(g\cdot K),$$ (6)

where *T* is the absolute temperature. One mole of polystyrene = 104.15 g.

Recently, accurate measurements were made of the specific heat capacity of polystyrene [27] of sample pieces cut from a different slab, 50 mm thick. The result was 2.0 percent higher [8,27] than that calculated by the use of Eq. (6). The result determined with the GW and the G calorimeter do not depend on knowing the specific heat capacity of graphite, because the temperature responses of those calorimeters are electrically calibrated. Therefore, these two results are considered to have less uncertainty than that determined with the PW calorimeter; and although this latter result is in reasonably good agreement with the three others shown, it is not included with the calorimetric calibration of the ^60^Co source.

Other comparisons of absorbed dose to water standards (calorimetric, Fricke dosimetry, and ionometric) are generally in agreement to about 1 percent. These are reported in Refs. [28–31].

### 7.2 H_2_-Saturated Results

**Fig. 27:**
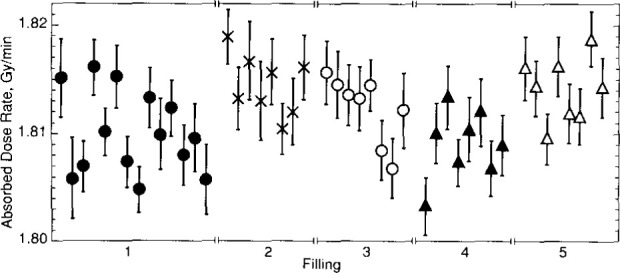
Daily averages of absorbed dose rate measurements with five fillings of water saturated with H_2_.

Figure 27 shows the results obtained with five fillings of water saturated with hydrogen. In nearly all cases, each point is the average of 32 measurements made in a day. The uncertainties are experimental standard deviations of the mean. Measurements for the first filling were made on 14 d. Measurements for the second, third, fourth, and fifth fillings were made on 8 d. The accumulated absorbed dose for each filling was about 1.25 kGy. Daily measurements began with or without a 30 min, 45 min, or 90 min pre-irradiation. On some days, a continuous irradiation period of 45 min or 90 min was applied after measurement No. 16 (midway of a daily set of measurements). In contrast to the N_2_-saturated water, the initial measurements in the H_2_-saturated water showed little or no initial exothermic effect.

**Fig. 28:**
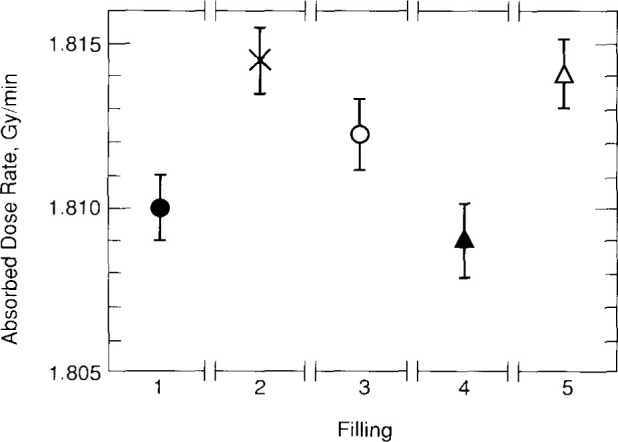
Average results of the five fillings saturated with H_2_.

**Fig. 29:**
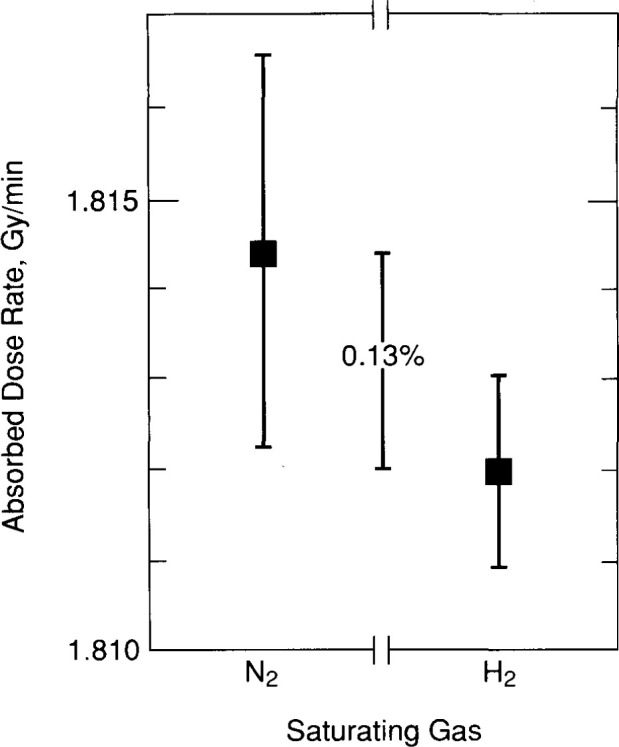
Comparison of the results of the N_2_- and H_2_-saturated systems.

A single value for each filling was calculated as the average of the points shown for that filling in Fig. 27. The results are shown in Fig. 28. The average value and the experimental standard deviation of the mean was calculated for those five points and are shown in Fig. 29. The results with the H_2_-and N_2_-saturated water are in good agreement and within the uncertainties. The 0.13 percent difference is in good agreement with, and in the same direction as the 0.3 percent difference reported by Klassen and Ross [20].

### 7.3 H_2_/O_2_-Saturated Results

**Fig. 30:**
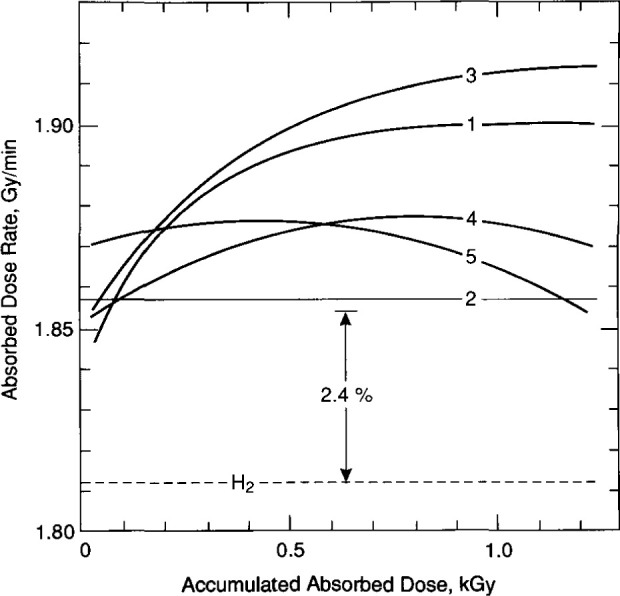
Measured results with five fillings of water saturated with mixtures of H_2_/O_2_ and comparison with the H_2_-saturated system.

Figure 30 shows the results with mixtures of H_2_/O_2_ and compared with the H_2_ result (from Fig. 29). The 2.4 percent exothermic effect is that reported [20] for saturating “high-purity” water with equal flow rates of H_2_ and O_2_, and where there was a large volume of those gases above the surface of *continuously circulated* water. The absorbed gases were quickly distributed throughout the water to keep their concentrations (and chemistry during irradiation) relatively constant. This is a different and relatively open system compared to the closed system described in this paper where only a small bubble of the gas remained in the glass detector assembly (Figs. 1 and 3) containing the *stagnant* water, a necessary requirement for measuring absorbed dose at a position. In such a system, the H_2_/O_2_ concentrations (and chemistry) change during irradiation.

In Fig. 30, the first filling shows that the initial exothermic effect is in the region of 2.4 percent, but increases further. The second filling shows a constant exothermic effect near 2.4 percent. The third filling shows the same general behavior as the first filling. The fourth filling has the same initial exothermic effect, but increases more slowly before showing a decrease. The fifth filling shows an initial exothermic effect of about 3 percent that increases slightly before slowly decreasing to about 2.4 percent at an accumulated absorbed dose of 1.2 kGy.

Subsequent to the measurements, a theoretical calculation was made for the H_2_/O_2_ closed system described as a function of accumulated absorbed dose and for different concentrations of the H_2_-and 02-saturating gases [32]. Subsequently, the gas flow rates measured with rotameters (Fig. 19) were checked with a more accurate method by use of a mass flowmeter [32] and by measuring the rate at which the gases displace a known volume of water. The flow rates as measured with the rotameters were significantly different. The tests showed that the detector assembly was saturated with a variable preponderance of H_2_ in the five fillings for the measurements of absorbed dose. The measured results as shown in Fig. 30 are, therefore, in general agreement with theory.

In retrospect, it was fortunate that the water was not saturated with equal flow rates of H_2_ and O_2_, because a preponderance of H_2_ “magnified” the variation of the heat defect with accumulated absorbed dose. The measurements, therefore, initiated the theoretical calculations [32] which led to the general conclusion that the closed H_2_/O_2_-saturated system is not recommended as a standard for measuring absorbed dose in *stagnant* water.

## 8. Corrections and Uncertainties

Negligible uncertainties were assigned to the following: (1) the specific heat capacity of water [22]; (2) measurements of source-detector distance and the 5 cm *linear* depth from the water surface to the horizontal plane where the thermistors were located, because of the uncertainty (~ 0.02 percent) in the optical sighting and the micrometer measurements; (3) measurements of the thermistor sensitivities; because of the reproducibility of their values before and after the experiment (from Table 1) and because the average of those values were used in the analysis; (4) beam exposure timing; and (5) excess heat generated in the temperature probes, because of the calculated heat flow analysis.

Ionization measurements in a water phantom showed that the aluminum foil and expanded polystyrene of the calorimeter lid decreased the measurements by 0.34 percent. This correction was made and a 0.05 percent uncertainty of one standard deviation was assigned. The effect of the thin glass wall was to cause the measurement depth to be slightly greater than if the wall had been “water equivalent.” A rough estimate based on ionization measurements and a simple geometry showed that the absorbed dose measurements had to be corrected by an increase of about 0.14 percent. A 0.1 percent estimated uncertainty was assigned.

The most difficult correction and uncertainty to assign is that related to the heat defect, if any. Figure 29 indicates that the difference of 0.13 percent is not significant for the N_2_ system, even though the measurements and theoretical calculations show that the H_2_ system is cleaner. The N_2_ system showed significant initial exothermic effects after a filling, which was not observed or relatively small for the H_2_ system. Also, there is good agreement with measurements made with the G and GW calorimeters, which have no heat defect. No correction is applied to the H_2_ system, but a 0.3 percent heat defect uncertainty is assigned. Table 2 lists the standard uncertainties with the H_2_ system. The combined standard uncertainty is 0.4 percent.

## 9. Conclusions and Future Plans

Based on the measurements and theoretical calculations, it appears that the H_2_-saturated system is the best practical choice in eliminating the heat defect in water. The flow rate of the gas need not be accurately known so long as there is an abundant supply (roughly about 30 cm^3^/min) bubbling through the water column such as shown in Fig. 19 for about 40 min. Measurements and tests indicate that the design of the calorimeter is sufficient for operation at room temperature.

Future plans are to significantly improve the operational efficiency by increasing the rapidity of making successive irradiation runs. This can be done by decreasing the time from the moment the water circulation is turned off to the time that the temperature drift is sufficiently small to start another irradiation run.

The motion and variation in temperature of the water in the region of the detector assembly was studied during the calibration of a thermistor removed from the glass container. Therefore, its response to the motion and temperature of the water was essentially instantaneous. About 10 min was required for the motion to subside. During this period there was a heat exchange between the water and its surroundings. This caused the water to be not strictly uniform in temperature. The variations in temperature could be significant compared to the small temperature rises during irradiation. It is required that the glass container be surrounded by motionless water so that if there is a temperature difference between that water and that within the container, heat conduction will take place to reduce the drifts to sufficiently small and predictable values during the irradiation period.

The above would require (1) a means for quickly stopping the motion of the water around the detector assembly after circulation, and (2) that the circulated water (immediately after power turn off) is essentially at the same temperature as that of the stagnant water in the detector assembly. The means for (1) would simply be a thin horizontal baffle positioned at some distance below the detector assembly and attached to its two supports (Fig. 1) and to investigate if the baffle does not significantly interfere with the water circulation. The means for accomplishing (2) is by making a slight modification in the Wheatstone bridge shown in Fig. 4. During circulation, a switch would replace R′ with a thermistor (not shown) in the circulated water, and replace thermistor 1 with a resistor. The bridge would then respond to temperature differences between the two bodies of water. This would indicate if the circulation needed to be continued or the water slightly heated with the immersed heaters (not shown) to produce a bridge null condition. This would reduce the time for the final drift to subside to small values.

## 10. Recommendation

A recommendation pointed out in Ref. [4] needs to be repeated concerning protection against significant variations in ambient temperature. Although this was not the case during the measurements, a structure was built for that purpose. It is a four-sided acrylic structure having an expanded polystyrene lid with a polyethylene film covering the beam entrance window. A temperature sensor, heater, and fan were mounted on an inside wall. The circulated air was controlled to about 0.02 °C and set to match that of the water in the calorimeter.

## Figures and Tables

**Table 1:** Thermistor material “constants,” *β*

Temperature Range (°C)	*β*_1_ (K)	*β*_2_ (K)
15–21–27	3206.64	3233.54
16–21–26	3206.93	3234.17
17–21–25	3206.30	3234.20
18–21–24	3205.92	3234.56
	3206.45 ± 0.007%	3234.12 ± 0.006%
16–22–28	3209.29	3237.01
17–22–27	3210.12	3236.57
18–22–26	3210.46	3236.83
19–22–25	3208.88	3236.55
	3209.69 ± 0.001%	3236.74 ±0.004%
17–23–29	3213.33	3239.68
18–23–28	3212.42	3239.78
19–23–27	3213.25	3238.94
20–23–26	3214.35	3239.00
	3213.34 + 0.012%	3239.35 ±0.007%
19–24–29	3216.57	3242.35
20–24–28	3215.56	3242.34
21–24–27	3217.35	3241.98
	3216.49 ±0.016%	3242.22 ± 0.004%
21–25–29	3220.47	3245.60
22–25–28	3218.70	3245.88
23–25–27	3221.26	3245.02
	3220.14 ± 0.024%	3245.50 ± 0.008%

**Table 2:** Uncertainties with the H_2_ system

Source	Estimated standard uncertainty (%)	
	Statistical, *s_i_* (degrees of freedom, *v_j_*)	Other, *μ_j_*
Heat defect		0.3
Reproducibility of measurement groups (Fig. 28)	0.15 (4)	
Beam attenuation from glass wall		0.1
Beam attenuation of calorimeter lid	0.05 (5)	

$\text{Combined standard uncertainty}=\sqrt{\Sigma s_{i}^{2}+\Sigma \mu_{j}^{2}}=0.4\%$

## Footnotes

1 Absorbed dose is the energy per unit mass imparted to matter at a point by ionizing radiation. The SI unit of absorbed dose is the gray (symbol Gy). 1 Gy=l J/kg (= 100 rad).
